# Retrospective Investigation in Horses with Encephalitis Reveals Unnoticed Circulation of West Nile Virus in Brazil

**DOI:** 10.3390/v14071540

**Published:** 2022-07-14

**Authors:** Hegger Fritsch, Felicidade Mota Pereira, Erica Azevedo Costa, Vagner Fonseca, Stephane Tosta, Joilson Xavier, Flavia Levy, Carla de Oliveira, Gabriela Menezes, Jaqueline Lima, Lenisa Santos, Luciana Silva, Vanessa Nardy, Marcela Kelly Gómez Astete, Beatriz Senra Álvares da Silva Santos, Nágila Rocha Aguiar, Maria Isabel Maldonado Coelho Guedes, Guilherme Canhestro de Faria, Ronaldo Furtini, Safira Rachel Milanez Drumond, Gabriel Muricy Cunha, Marcia São Pedro Leal Souza, Ronaldo de Jesus, Sara A. Franco Guimarães, Italo Coelho Nuno, Ian Carlos Brito de Santana, José Eduardo Ungar de Sá, George Roma Santos, Willadesmon Santos Silva, Thiago Ferreira Guedes, Emerson Luiz Lima Araújo, Rodrigo Fabiano do Carmo Said, Carlos Frederico Campelo de Albuquerque, Cassio Roberto Leonel Peterka, Alessandro Pecego Martins Romano, Rivaldo Venâncio da Cunha, Ana Maria Bispo de Filippis, Arabela Leal e Silva de Mello, Marta Giovanetti, Luiz Carlos Junior Alcantara

**Affiliations:** 1 Universidade Federal de Minas Gerais, Belo Horizonte 31270-901, Brazil; hegger.fritsch@gmail.com (H.F.); azevedoec@yahoo.com.br (E.A.C.); sttosta@gmail.com (S.T.); joilsonxavier@live.com (J.X.); beatrizsenra.santos@gmail.com (B.S.Á.d.S.S.); naaguilar@hotmail.com (N.R.A.); mariaisabel.guedes@gmail.com (M.I.M.C.G.); 2 Laboratório Central de Saúde Pública Prof Goncalo Moniz, Salvador 41745-900, Brazil; felicidade.pereira@saude.ba.gov.br (F.M.P.); gabrielaa.smenezes@gmail.com (G.M.); jackgomes_@hotmail.com (J.L.); dandarassa@hotmail.com (L.S.); roboredo.oliveira@gmail.com (L.S.); vanessanardy@gmail.com (V.N.); astetegomezmarcelak@gmail.com (M.K.G.A.); sarafrancoguimaraes@gmail.com (S.A.F.G.); italoracema@outlook.com (I.C.N.); ian_santana@outlook.com (I.C.B.d.S.); veterfarmac@gmail.com (J.E.U.d.S.); georgedudananda@gmail.com (G.R.S.); willadesmon.silva@saude.ba.gov.br (W.S.S.); 3 Organização Pan-Americana de Saúde/Organização Mundial de Saúde, Brasilia 37650-000, Brazil; vagner.fonseca@gmail.com (V.F.); saidrod@paho.org (R.F.d.C.S.); meloc@paho.org (C.F.C.d.A.); 4 Laboratorio de Flavivirus, lnstituto Oswaldo Cruz/Fundação Oswaldo Cruz, Rio de Janeiro 21040-360, Brazil; flaviallevy@yahoo.com.br (F.L.); oliveirasc@yahoo.com.br (C.d.O.); ana.bispo@ioc.fiocruz.br (A.M.B.d.F.); 5 Laboratório de Saúde Animal, Instituto Mineiro de Agropecuária, Belo Horizonte 30110-005, Brazil; lsa@ima.mg.gov.br (G.C.d.F.); ronaldoanas@ig.com.br (R.F.); srmdrumond@hotmail.com (S.R.M.D.); 6 Secretary of Health of the State of Bahia (SESAB), Salvador 40301-110, Brazil; cunha.gabrielmuricy@gmail.com (G.M.C.); marciaspls@yahoo.com.br (M.S.P.L.S.); 7 Coordenação Geral dos Laboratórios de Saúde Pública, Secretaria de Vigilância em Saúde-Brazil, Brasília 70719-040, Brazil; ronaldo.jesus@saude.gov.br (R.d.J.); thiago.guedes@saude.gov.br (T.F.G.); emerson.araujo@saude.gov.br (E.L.L.A.); 8 Coordenação Geral das Arboviroses, Secretaria de Vigilância em Saúde (CGARB/SVS-MS), Brasilia 37650-000, Brazil; carlos.peterka@saude.gov.br (C.R.L.P.); alessandropecego@gmail.com (A.P.M.R.); 9 Fundação Oswaldo Cruz, Bio-Manguinhos, Rio de Janeiro 21040-360, Brazil; rivaldo.rivaldo.cunha@fiocruz.br; 10 Department of Science and Technology for Humans and the Environment, University of Campus Bio-Medico, 00128 Rome, Italy

**Keywords:** WNV, nanopore sequencing, genomic monitoring, Brazil

## Abstract

During these past years, several studies have provided serological evidence regarding the circulation of West Nile virus (WNV) in Brazil. Despite some reports, much is still unknown regarding the genomic diversity and transmission dynamics of this virus in the country. Recently, genomic monitoring activities in horses revealed the circulation of WNV in several Brazilian regions. These findings on the paucity of genomic data reinforce the need for prompt investigation of WNV infection in horses, which may precede human cases of encephalitis in Brazil. Thus, in this study, we retrospectively screened 54 suspicious WNV samples collected between 2017 and 2020 from the spinal cord and brain of horses with encephalitis and generated three new WNV genomes from the Ceará and Bahia states, located in the northeastern region of Brazil. The Bayesian reconstruction revealed that at least two independent introduction events occurred in Brazil. The first introduction event appears to be likely related to the North American outbreak, and was estimated to have occurred in March 2013.The second introduction event appears to have occurred in September 2017 and appears to be likely related to the South American outbreak. Together, our results reinforce the importance of increasing the priority of WNV genomic monitoring in equines with encephalitis in order to track the dispersion of this emerging pathogen through the country.

## 1. Introduction

West Nile virus (WNV) is an arthropod-borne pathogen and a member of the Flaviviridae family, belonging to the Japanese encephalitis serocomplex [1]. WNV was first isolated in 1937, in the West Nile district of Uganda, causing a low-grade febrile illness [2]. Afterwards, this virus was detected in the Middle East, Europe, Australia, Africa and South Asia [1,2]. Since its first introduction in North America in 1999 and its fast dispersion through South America [3,4], much serological evidence of WNV was reported, suggesting a potential spill-over into human populations and new outbreaks.

The circulation of WNV is maintained in nature by its high viremia in birds and mosquitoes, particularly in *Culex pipiens* and *Cx. quinquefasciatus* species [5,6]. Despite the bird-mosquito cycle, WNV can also infect humans and other mammals, such as equines, as “dead-end” hosts, given that the low viremic infection is insufficient to contribute to WNV spread via the mosquito bite [5,6]. Regardless of its potential for causing neuroinvasive symptoms, around 80% of WNV infections in humans are asymptomatic, whereas the remaining cases may develop mild or severe manifestations. The most common clinical symptoms include fever, headache, tiredness and vomiting [7,8]. Due to the presence of comorbidities and immunological disorders, some patients can show more severe complications, which can lead to high fever, coma, convulsions and paralysis [7,8]. When infected, equines occasionally manifest neurological disease and death [7,8], serving as a sentinel population for WNV outbreaks and enabling transmission to humans.

Although the first detection of WNV in South America occurred in Argentina (2006) when the virus was isolated from a horse with encephalitis, the first genome sequenced in Brazil was obtained only by the end of 2018. Despite little serological evidence and reports of WNV infections in humans and other animals [9,10,11,12,13,14,15,16,17,18], much is still unknown regarding the genomic diversity, evolution and transmission dynamics of this virus in the country. Recently, genomic monitoring activities in horses revealed the circulation of WNV in several states located in southeastern and northeastern Brazil [19,20]. The lack of genomic data, in addition to the presence of a potential factor for the unnoticed circulation of WNV in Brazil, reinforces the need for prompt investigation of WNV infection in horses, which may precede human cases of encephalitis. Thus, in this study, we retrospectively screened 54 suspicious WNV samples collected between 2017 to 2020 from horses with encephalitis and generated two new WNV genomes from the northeastern region.

## 2. Materials and Methods

### 2.1. Ethical Statement

This research was reviewed and approved by the Ethical Committee of the Pan American World Health Organization (No. PAHO-2016-08-0029), the Oswaldo Cruz Foundation Ethics Committee (CAAE: 90249218.6.1001.5248), the Ethical Committee of the Federal University of Minas Gerais (UFMG) (CAAE: 32912820.6.1001.5149) and the Brazilian Ministry of Health (MoH) as part of the arbovirus genomic surveillance efforts within the terms of Resolution 510/2016 of CONEP (Comissão Nacional de Ética em Pesquisa, Ministério da Saúde; National Ethical Committee for Research, Ministry of Health).

### 2.2. Sample Collection and RNA Isolation

Samples (brain and spinal cord) from 54 horses with encephalitis symptoms collected between 2017 and 2020 from northeastern Brazil were sent for molecular diagnosis for rabies virus at the Laboratório Central de Saúde Pública (Professor Gonçalo Moniz; LACEN-BA, Salvador, Bahia). The viral RNAs were extracted using the QIAamp Viral RNA Mini Kit (Qiagen, Hilden, Germany) according to the manufacturer’s instructions.

### 2.3. cDNA and Multiplex Sequencing PCR Procedures

All suspected WNV samples were submitted to a cDNA synthesis protocol [19,21] using the SuperScript IV cDNA Synthesis Kit. Then, a whole-genome multiplex PCR was performed in order to amplify the entire coding region of WNV using two primer pools (A and B) in separated tubes. Reaction and thermocycling conditions were previously described by [19,21].

### 2.4. Library Preparation and Viral Whole-Genome Nanopore Sequencing

After the PCR, the amplified products were purified using 1× AMPure XP Beads (Beckman Coulter, Brea, CA, USA) and the DNA concentration was quantified using the Qubit dsDNA HS Assay Kit on the Qubit 3.0 Fluorometer (Thermo Fisher, Waltham, MA, USA). DNA library preparation was conducted for the two samples, which presented DNA concentrations >1 ng/uL after the clean-up procedure. For that, the Ligation Sequencing Kit (Oxford Nanopore Technologies, Oxford, UK) and Native Barcoding Expansion 96 Kit (Oxford Nanopore Technologies) were used following the reaction conditions previously described in [21]. The sequencing library was generated using SQK-LSK109 (Oxford Nanopore Technologies) and loaded onto a R9.4 flow cell (Oxford Nanopore Technologies). Sequencing was performed for 8 h on a MinION device and the raw data were processed for basecalling and demultiplexing using the Guppy software. The final consensus sequences were obtained using the Genome Detective software (https://www.genomedetective.com/ accessed on 30 June 2022) [22].

### 2.5. WNV Lineage Classification

The WNV lineage assignment was confirmed in a two-step procedure: (1) using the Genome Detective West Nile Virus Typing Tool (available at: https://www.genomedetective.com/app/typingtool/wnv; accessed on 22 April 2022); and (2) through conducting a maximum likelihood (ML) phylogenetic analysis.

For this purpose, we retrieved a total of 2152 genome sequences with associated lineage date and country of collection from GenBank, collected up to April 2022 (Supplementary Table S1). Alignment was performed using MAFFT [23] and manually curated to remove artifacts using AliView [24]. The maximum likelihood (ML) phylogenetic tree was estimated using IQ-TREE [25] under the HKY + G4 nucleotide substitution model, which was inferred as the most accurate evolutionary model in jModelTest2 (https://github.com/ddarriba/jmodeltest2 accessed on 30 June 2022) [26]. The robustness of the tree topology was determined using 1000 bootstrap replicates. The ML tree was visualized and edited using FigTree v.1.4.4 (https://github.com/rambaut/figtree/releases accessed on 30 June 2022).

### 2.6. Phylogenetic and Bayesian Analysis

In order to explore the relationship between the new genomes obtained in this study and other isolated sequences from this dataset, we generated a subset that included the highly supported (>0.9) clade containing the new WNV strains obtained in this study plus 26 global reference sequences (Supplementary Table S2). The phylogenetic signal was assessed by using the likelihood mapping approach implemented in the IQ-TREE2 version [26]. A new ML tree was reconstructed in the IQ-TREE2 software, under the HKY + G4 substitution model [26].

Prior to the Bayesian analysis, this subset was also assessed for a molecular clock signal using the root-to-tip regression method available in TempEst v1.5.3 [27] following the removal of potential outliers that may violate the molecular clock assumption. Time-scaled phylogeny was then inferred using the BEAST v1.10.4 package [28] under the uncorrelated relaxed molecular clock model and the nonparametric Bayesian Skyline coalescent model. We computed MCMC (Markov chain Monte Carlo) duplicate runs of 10 million states each, sampling 1000 steps. Convergence of MCMC chains was checked using Tracer v.1.7.2 [29]. The maximum clade (MC) tree was summarized using TreeAnnotator (http://beast.community/index.html accessed on 30 June 2022) with 10% of burn-in.

## 3. Results

**Figure 1:**
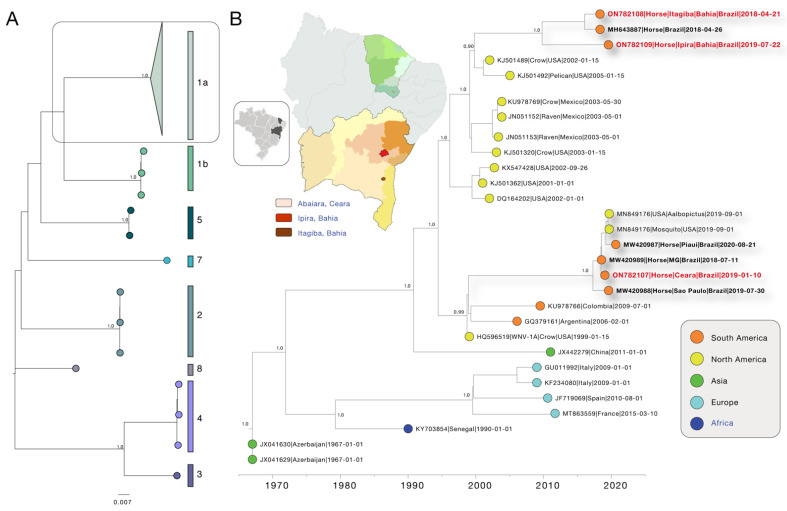
Genomic monitoring of WNV in northeastern Brazil. (**A**) ML reconstruction showing major linages of WNV, including n = 2152 genome sequences plus the three new strains obtained from this study. The scale bar is in units of substitutions per site (*s*/*s*). Support for the branching structure is shown by bootstrap values at nodes. The highlight indicates that the new genomes belong to the 1a lineage. (**B**) Map of Brazilian Northeast Region, highlighting the states of Bahia and Ceará. Time-scaled maximum clade credibility phylogeny of WNV, including the two new genomes generated in this study plus *n* = 26 reference strains. Tips are colored according to the sampling location. Values around key nodes represent posterior probability support.

To investigate the circulation of WNV in the Brazilian Northeast Region, we generated two new genomes using nanopore sequencing. Phylogenies estimated by the WNV typing tool, along with maximum likelihood methods, consistently placed the Brazilian genomes in a single clade within the 1a lineage with maximum statistical support (bootstrap = 100%) (Figure 1A). The newly generated samples were collected, between 2018 and 2019, from three equids that presented neurological manifestations. Those samples were isolated in the states of Bahia and Ceará (Figure 1B), located in northeastern Brazil.

After sequencing, the consensus sequences presented an average genome coverage of 92.95% (91.6 and 94.3%). Sample 01 (ON782108) from 21 April 2018 was collected from an 8-year-old female horse in the city of Itagibá, Bahia. The second sample (Sample 02—ON782109) from 22 July 2019 was sampled from a 4-year-old male horse obtained in the location of Ipirá, Bahia (Figure 1B). Sample 03 (ON782107) from 5 January 2019 was collected from a 4-year-old female horse, obtained in Abaiara, Ceará, which was showing a neurological disorder as well as neuromotor symptoms and behavior impairment. For samples 01 and 02, it was not possible to recover the equids’ clinical manifestations. Epidemiological information and sequencing statistics of the three sequenced samples of WNV are showed in Table 1.

To establish the relationship between the new WNV genome sequences obtained in this study and other isolates from Brazil and other closely related countries, a combined dataset was subjected to Bayesian phylogenetic inference. A regression of genetic divergence from root to tip against sampling dates confirmed a sufficient temporal signal (coefficient correlation = 0.83, r^2^ = 0.70). Our maximum clade credibility (MCC) tree suggested that Brazil faced two independent introduction events, as indicated by two distinct clades (Figure 1C). Additionally, it was also possible to observe that the sample collected in the state of Ceará clustered together with isolates collected in other Brazilian regions (Northeast and Southeast), suggesting that the states of São Paulo, Minas Gerais and Piauí must be likely involved in the same transmission route (Figure 1C). The second cluster includes the new isolates obtained from the state of Bahia (northeastern Brazil) and an isolate from the state of Espírito Santo, which is located in the southeastern region. From our time-measured tree, we estimated the time of the most recent common ancestor (TMRCA) of the WNV epidemic in Brazil to have occurred around early March 2013 (95%HPD: late September 2002 to mid-May 2015) for the first introduction event, and, more recently, early September 2017 (95%HPD: late April 2012 to late June 2018) for the second event (Figure 1C). The presence of clustered samples from 2018 to 2019 and 2018 to 2020 in cluster one and two, respectively, revealed a cryptic and persistent transmission in those states and within the country. The estimated dates of introduction further indicate the duration of the pre-detection cryptic transmission in those recipient regions, highlighting how crucial the strength of genomics monitoring of this viral pathogen is to prevent its spread and reduce its epidemic potential in the region.

## 4. Discussion

In this study, using portable nanopore sequencing, we generated three new WNV-1a genomes collected between 2017 and 2019 from the northeastern region of Brazil. Among the zoonotic arboviruses detected in Brazil [14,30,31,32,33,34,35,36,37,38,39], since 2004, several studies have provided serological evidence regarding the circulation of West Nile virus (WNV) in mammal and bird reservoirs [10,14,15,16,17,18,19,20,40,41,42]. Even though the circulation of WNV has been documented since 2004, the short period of infection, followed by a low viremia and the difficulty of accessing the possible infected animals reduced the possibility of molecular confirmation and sequencing [9,10,14,15,16,17,18,20,40,41,42]. The lack of genomic data and viral surveillance decreases the possibility of reconstructing the dispersal routes of this pathogen and monitoring sources of new introductions.

Our data reinforce the idea that multiple independent introduction events of WNV have occurred in the country, which also appears to be in line with recently released data [19]. Our data suggest that the first importation events, likely related to the North America outbreak, occurred in early March 2013, whereas the second introduction, likely related to the circulation of WNV in South America (potentially in Colombia), occurred in early September 2017. Despite our data suggesting the earliest introduction may have occurred in 2013, the reporting of WNV circulation in mammals and birds since 2004, as shown in several serological studies [10,14,15,16,17,18,19,20,40,41,42], might reflect a possible cryptic transmission of this virus in the region. This hypothesis is also corroborated by the detection of WNV in an equid sampled in Boa Viagem, Ceará in 2019. These two detections suggest the maintenance of WNV circulation in this state [20]. The absence of correspondence between the serological evidence and the genomic data is a reflection of the lack of genomic surveillance which hampers our abilities to infer an accurate estimate of introduction.

Between 2017 and 2019, 715 arboviral neuroinvasive cases in humans were reported in Brazil [43]. Although the incidence of severe complications remains low when compared to the number of arboviruses cases [44,45], neurological disorders are increasing [43]. To date, the Brazilian Ministry of Health notified 165 cases of WNV infection between 2017 and 2021. Regarding WNV surveillance, the monitoring of wild birds and equines with neurological symptomatology has been adopted as a strategy for detecting the presence of the virus in Brazil. Although the data are still scarce, we can assume that West Nile virus might be circulating unnoticed throughout the national territory [20]. Together, our results reinforce the importance of strengthening epidemiological and genomics investigations towards zoonotic arbovirus in Brazil, increasing the priority of WNV genomic surveillance in equines with encephalitis in order to follow the dispersion of this emerging pathogen through the country.

## Figures and Tables

**Table 1:** Epidemiological information and sequencing statistics of the three sequenced samples of WNV sampled in the Brazilian states of Bahia and Ceará.

Acession Number	Lineage	Sample Type	Collection Date	State	Municipality	Sex	Age	Reads	Coverage (%)	Clinical Sign
ON782108	1a	Brain/Spinal cord	21 April 2018	Bahia	Itagibá	Female	8	179,788	91.6	NA
ON782109	1a	Brain/Spinal cord	22 July 2019	Bahia	Ipirá	Male	4	260,093	94.3	NA
ON782107	1a	Brain	10 January 2019	Ceará	Abaiara	Female	4	290,093	88.9	Neurologic disorder and neuromotor impairment

## Data Availability

Newly generated WNV sequences were deposited in GenBank under accession numbers ON782108, ON782109 and ON782107.

## Supplementary Materials

The following supporting information can be downloaded at: https://www.mdpi.com/article/10.3390/v14071540/s1, Table S1: List of all reference sequences used for the lineage assignment (*n* = 2152); Table S2: List of all reference sequences used in the time-scaled Bayesian.
